# 4227 Closing the cross-institutional referral loop: Applying human factors to improve consultations

**DOI:** 10.1017/cts.2020.171

**Published:** 2020-07-29

**Authors:** April Savoy, Michael Weiner, Teresa Damush

**Affiliations:** 1 Indiana University-Purdue University at Indianapolis

## Abstract

OBJECTIVES/GOALS: Although referrals for specialty consultations are a core clinical process, they are prone to coordination and communication breakdowns that have led to adverse clinical outcomes. This project’s objective is to improve timely documentation, transmission, access, and quality of consultation notes across healthcare systems. METHODS/STUDY POPULATION: There are two specific aims for this project. In Aim 1, we will characterize clinical workflows and information flow during cross-institutional referrals. In Aim 2, we will develop and test a prototype leveraging electronic health information exchange (HIE) to increase closing the loop for cross-institutional referrals and improve the quality of consultation notes. To accomplish these aims, we will use human factors methods, including data analytics, medical-record reviews, semi-structured interviews of consultants, rapid prototyping, and usability evaluations. RESULTS/ANTICIPATED RESULTS: Results will inform the design and integration of clinician-facing technologies into clinical workflows to close the referral loop and improve diagnostic processes. Aim 1 will provide quantitative evidence about the quality of cross-institutional referrals, inform the eventual implementation of our prototype, and identify user interface features required for successful electronic health information exchange. Based on the results from Aim 1, reports and visual representations will be generated to illustrate information flows and clinical workflows. This will prioritize design efforts for the intervention’s prototype. Aim 2 will translate clinicians’ requirements into prototype features and assess clinicians’ experience with the prototype. DISCUSSION/SIGNIFICANCE OF IMPACT: The use and usefulness of HIE has been limited due to usability and implementation issues. Cross-institutional referrals are complex and dependent on HIE due to EHRs’ lack of interoperability. This project will provide evidence-based recommendations for the use of Fast Healthcare Interoperability Resources (FHIR) to improve HIE during referrals.

